# The novel oligopeptide utilizing species *Anaeropeptidivorans aminofermentans* M3/9^T^, its role in anaerobic digestion and occurrence as deduced from large-scale fragment recruitment analyses

**DOI:** 10.3389/fmicb.2022.1032515

**Published:** 2022-11-09

**Authors:** Irena Maus, Daniel Wibberg, Peter Belmann, Sarah Hahnke, Liren Huang, Cathrin Spröer, Boyke Bunk, Jochen Blom, Alexander Sczyrba, Alfred Pühler, Michael Klocke, Andreas Schlüter

**Affiliations:** ^1^Genome Research of Industrial Microorganisms, Center for Biotechnology (CeBiTec), Bielefeld University, Bielefeld, Germany; ^2^Computational Metagenomics, Forschungszentrum Jülich GmbH, Institute of Bio- and Geosciences IBG-5, Jülich, Germany; ^3^Faculty of Technology, Bielefeld University, Bielefeld, Germany; ^4^Department of Human Medicine, Carl von Ossietzky Universität Oldenburg, Oldenburg, Germany; ^5^Department Bioinformatics and Databases, Leibniz Institute DSMZ-German Collection of Microorganisms and Cell Cultures GmbH, Brunswick, Germany; ^6^Department Bioinformatics and Systems Biology, Justus-Liebig University Giessen, Giessen, Germany; ^7^Institute of Agricultural and Urban Ecological Projects affiliated to Humboldt-Universität zu Berlin (IASP), Berlin, Germany

**Keywords:** metagenome, plant biomass hydrolysis, renewable energy, *Lachnospiracea*e, omics analyses

## Abstract

Research on biogas-producing microbial communities aims at elucidation of correlations and dependencies between the anaerobic digestion (AD) process and the corresponding microbiome composition in order to optimize the performance of the process and the biogas output. Previously, *Lachnospiraceae* species were frequently detected in mesophilic to moderately thermophilic biogas reactors. To analyze adaptive genome features of a representative *Lachnospiraceae* strain, *Anaeropeptidivorans aminofermentans* M3/9^T^ was isolated from a mesophilic laboratory-scale biogas plant and its genome was sequenced and analyzed in detail. Strain M3/9^T^ possesses a number of genes encoding enzymes for degradation of proteins, oligo- and dipeptides. Moreover, genes encoding enzymes participating in fermentation of amino acids released from peptide hydrolysis were also identified. Based on further findings obtained from metabolic pathway reconstruction, M3/9^T^ was predicted to participate in acidogenesis within the AD process. To understand the genomic diversity between the biogas isolate M3/9^T^ and closely related *Anaerotignum* type strains, genome sequence comparisons were performed. M3/9^T^ harbors 1,693 strain-specific genes among others encoding different peptidases, a phosphotransferase system (PTS) for sugar uptake, but also proteins involved in extracellular solute binding and import, sporulation and flagellar biosynthesis. In order to determine the occurrence of M3/9^T^ in other environments, large-scale fragment recruitments with the M3/9^T^ genome as a template and publicly available metagenomes representing different environments was performed. The strain was detected in the intestine of mammals, being most abundant in goat feces, occasionally used as a substrate for biogas production.

## Introduction

Microbiomes are broadly applied in a variety of biotechnological processes, as example, for the generation of bio-energy by anaerobic digestion (AD) of residual biomass ultimately resulting in biogas (Koch et al., 2014). Due to tremendous advances in the development of High-Throughput (HT) sequencing technologies, microbiomes are now widely studied using various methods of metagenome research. Deep sequencing of metagenomes from biogas microbiomes resulted in the reconstruction of genomes from microbial community members enabling the analysis of microorganisms difficult or currently impossible to cultivate (Stolze et al., 2016; Kougias et al., 2018; Maus et al., 2020a). Thousands of Metagenomically Assembled Genomes (MAGs) have been compiled (Campanaro et al., 2020). Hereby, MAG-based metabolic reconstruction can provide substantial insights into the metabolic potential of microorganisms allowing conclusions on their functional role in microbial ecosystems.

Unfortunately, the experimental verification of metabolic features and/or ecological function derived from MAG analysis is still hampered by the limitations in the cultivation of microbial species of interest. The percentage of microbial species that are accessible by conventional laboratory cultivation methods was estimated to be in the range of 0.1 to 1% (Solden et al., 2016). In consequence, the combination of genomics with comprehensive cultivation strategies (culturomics) are proposed as essential to provide the basis for the experimental accessibility of AD microbiome members (Greub, 2012; Lagier et al., 2012, 2015).

Up-to-date readily cultivated type strains for bacteria participating in AD are i.a. members of the phyla *Actinobacteria*, *Bacteroidetes*, *Chloroflexi*, *Deferribacteres*, *Bacillota* (syn. *Firmicutes*), *Lentisphaerae*, *Nitrospira*, *Proteobacteria*, *Synergistetes*, and *Thermotogae*. Due to the importance of cellulolytic *Clostridia* (phylum *Bacillota*) in plant biomass degradation, their isolation was in the focus of attention during the last years leading to the description of several new cellulolytic genera, e.g., *Herbinix* (*Lachnospiraceae*, *Bacillota*) (Koeck et al., 2015, 2016a) and *Herbivorax* (*Ruminococcaceae*, *Bacillota*) (Koeck et al., 2016b). In addition, species featuring anaerobic proteolytic activity were isolated from anaerobic digesters, and their genomes were completely sequenced. For example, protein or oligopeptide degrading species of the genera *Fermentimonas*, *Proteiniphilum*, and *Petrimonas* were isolated and physiologically characterized (Hahnke et al., 2016; Maus et al., 2020a).

The last step in AD but most crucial for bio-energy production is the methanogenesis accomplished by highly specialized *Archaea*. Recently, genomes of methanogenic species from the genera *Methanobacterium*, *Methanobrevibacter*, *Methanothermobacter*, *Methanocorpusculum*, *Methanoculleus*, *Methanolinea*, *Methanoregula*, *Methanospirillum*, *Methanothrix*, and *Methanosarcina* were released in public databases (Lambie et al., 2015; Maus et al., 2015; Tejerizo et al., 2017; Hassa et al., 2019).

For further biotechnological improvements and applications, a deeper understanding of primary hydrolysis of high-molecular biomass compounds, such as proteins, is commonly assumed as an indispensable pre-requirement. In this context, the potential of the family *Lachnospiraceae* as an important member of the mammalian and human intestine core microbiome (Sorbara et al., 2020; Vacca et al., 2020) is widely unexploited. *Lachnospiraceae* have an anaerobic fermentative metabolism and produce short-chain fatty acids such as acetate, propionate, and butyrate. Some species of the *Lachnospiraceae* are able to hydrolyze carbohydrates and polysaccharides, others decompose proteinaceous substrates, or both (Cai and Dong, 2010; Sorbara et al., 2020; Vacca et al., 2020). *Lachnospiraceae* bacteria end up in biogas digesters with the residues from livestock farming used as feedstock in the biogas fermentation process. Currently, the Genome Taxonomy Database (GTDB; Chaumeil et al., 2020) comprises about 4,000 entries assigned to the family *Lachnospiraceae* (December 2021). Only a small fraction of the corresponding MAGs is represented by isolates.

As example, *Anaerotignum aminivorans* DSM 103575^T^ was isolated from a methanogenic reactor treating manure/excrement from livestock farming (Ueki et al., 2017). Likewise, *Anaerotignum neopropionicum* DSM 3847^T^ (formerly *Clostridium neopropionicum* X4) was isolated from a mesophilic industrial reactor operated for fermentation of vegetable cannery wastewater (Beck et al., 2016). Further strains of the genus *Anaerotignum* such as *A. faecicola* DSM 107953^T^ (Choi et al., 2019), *A. propionicum* DSM 1682^T^ (Poehlein et al., 2016) and the lactate-fermenting bacterium *Anaerotignum lactatifermentans* DSM 14214^T^ (van der Wielen et al., 2002; Ueki et al., 2017) were isolated from human feces, black mud of marine sediment and from the caeca of a chicken, respectively. All currently described *Anaerotignum* isolates share common metabolic characteristics directed towards the utilization of proteins, oligopeptides, and in particular amino acids. Accordingly, members of this *Lachnospiraceae* genus seem to complement the function of cellulolytic and carbohydrate-decomposing clostridial species in biogas microbiomes.

Closely related to the genus *Anaerotignum* is the recently newly proposed genus *Anaeropeptidivorans* (Köller et al., 2022), represented by the type strain *Anaeropeptidivorans aminofermentans* M3/9^T^ (Köller et al., 2022), which was obtained from a laboratory-scale biogas reactor. Due to the close affiliation with *Anaerotignum*, proteolytic functioning in AD systems is hypothesized also for *A. aminofermentans.* In this study, the complete genome of *A*. *aminofermentans* M3/9^T^ was sequenced and analyzed in detail to reconstruct its phylogenetic relationships within the family *Lachnospiraceae* and to address the identification of genetic determinants potentially specifying competitiveness of *Anaeropeptidivorans* species in AD environments. Moreover, compilation of the *A. aminofermentans* M3/9^T^ genome sequence enabled determination of its occurrence in AD microbiomes by applying metagenome fragment recruitments thereby opening perspectives in relation to microbiome-based management of the biogas process.

## Materials and methods

### Strain isolation, cultivation and DNA extraction

Strain M3/9^T^ was obtained from a mesophilic two-phase biogas reactor, i.e., upflow anaerobic solid-state (UASS) reactor (Mumme et al., 2010), continuously supplied with a mixture of 95% maize silage and 5% wheat straw as described previously (Köller et al., 2022). Briefly, the reactor was operated at an organic loading rate of 3 g VS_corr_ l^−1^ d^−1^, whereby VS_corr_ was defined as the sum of volatile solids, volatile fatty acids, lactic acid, and alcohols. Strain M3/9^T^ was isolated from 10-fold diluted reactor fluid on BBL™ Columbia Agar Base medium (Becton Dickinson) supplemented with 5% laked horse blood. For purification, single colonies were picked and restreaked; incubation occurred at 37°C.

To obtain biomass for DNA extraction, the strain was cultivated at 37°C under a 100% N_2_ gas atmosphere in Tryptic Soy Broth/Casein-peptone Soymeal-peptone Broth (Merck) supplemented with 2 g l^−1^ yeast extract. Subsequent extraction of genomic DNA was performed using the Gentra Puregene Yeast/Bact. Kit (Qiagen, Hilden, Germany) following the manufacturer’s instructions. The obtained DNA was purified using the NucleoSpin® gDNA Clean-up kit (Macherey-Nagel, Düren, Germany) for sequencing purposes.

### PacBio library preparation, sequencing, genome assembly and annotation

The construction of the PacBio library, followed by the PacBio sequencing and genome assembly were performed as described previously (Nelkner et al., 2019; Maus et al., 2020b). In brief, extracted DNA was treated with the Zymo Genomic DNA Clean & ConcentratorTM-10 kit. The SMRTbell™ template library was prepared following the’Procedure & Checklist – Greater Than 10 kb Template Preparation’ from PacificBiosciences (Menlo Park, CA, United States). SMRT sequencing was carried out on the PacBio RSII (PacificBiosciences, Menlo Park, CA, United States) at DSMZ taking one 240-min movie for one SMRT cell using the P6 Chemistry. Sequencing resulted in 60,057 post-filtered reads with a mean read length of 7,645 bp. SMRT Cell data was assembled using the “RS_HGAP_Assembly.3″ protocol within the SMRT Portal version 2.3.0 using default parameters. Assembly validation was performed using the “RS_Bridgemapper.1″ protocol. The chromosome was circularized and adjusted to *dna*A as the first gene.

The genome annotation and visualization were accomplished applying the GenDB 2.0 platform (Meyer et al., 2003) and PROKKA 1.14.5 (Seemann, 2014). Additionally, the genome was screened for genomic island regions, phages, pathogen-associated genes, virulence factors, and antibiotic resistance genes with IslandViewer 4 (Bertelli et al., 2017), PHASTER (Arndt et al., 2016) and CARD (Comprehensive Antibiotic Resistance Database (Jia et al., 2017)).

### Phylogenetic analysis and deduction of metabolic pathways

The finished genome sequence was imported into the annotation platform GenDB (Meyer et al., 2003) for the automatic prediction of genes as described previously (Tomazetto et al., 2018). The program EDGAR 3.0 (Dieckmann et al., 2021), a software tool for the comparative analysis of prokaryotic genomes, was applied to reconstruct *Lachnospiraceae* phylogeny as well as to perform comparative genome analysis of *Lachnospiraceae* species. The carbohydrate-active enzyme database (CAZy) annotation web-server dbCAN (Zhang et al., 2018) was applied to predict genes encoding carbohydrate-active enzymes. To predict peptidases encoded in the M3/9^T^ genome, all proteins were compared against the MEROPS database (Rawlings et al., 2018). Obtained hits with an e-value of >1×10^−50^ were discarded, with 118 hits remaining. The latter were compared against the PFAM database leading to 69 hits with an e-value of <1×10^−10^ for further characterization. Metabolic pathways of interest were reconstructed based on EC numbers of enzymes and their assignment to KEGG pathway maps.^1^

### Fragment recruitment

To determine the occurrence and abundance of *A. aminofermentans* M3/9^T^ in possible other metagenomic environments, large-scale fragment recruitment was performed using the genome sequences of M3/9^T^ and four closely related *Anaerotignum* members in 477,981 data sets (Supplementary Table S1) of the Sequence Read Archive (SRA) Mirror of the de.NBI Cloud.^2^ The search has been done by first pre-filtering all metagenomic SRA data sets applying Mash Screen (version: quay.io/biocontainers/mash:2.3--he348c14_1, Ondov et al., 2019) using “-w” parameter. In a second step, pre-selected data sets were aligned using BWA (version: quay.io/biocontainers/bwa:0.7.17--pl5.22.0_2, Li, 2013) using default parameters against all genomes in order to construct an alignments. The resulting alignments were finally analyzed using CoverM 0.6.1^3^ and the parameters “--methods trimmed_mean covered_bases relative_abundance trimmed_mean covered_fraction” for all genomes and the parameter “--min-read-percent-identity 95” for the M3/9^T^ genome. Corresponding computations were scaled-up and parallelized in the de.NBI Cloud by using Nextflow 21.10.5 (Di Tommaso et al., 2017) and the SimpleVM Cluster Mode which is based on Bibigrid.^4^ Fragment Recruitment plots were generated using R 3.6.3 and ggplot2 3.3.5.

### Analysis of fermentation products

For analysis of fermentation products, the strain was grown at 37°C under a 100% N_2_ gas atmosphere in Brain Heart Infusion Broth (Carl Roth) supplemented with 0.4 g l^−1^ yeast extract. Medium without addition of cells was used as control, assays were carried out in triplicates. Hydrogen (H_2_) and carbon dioxide (CO_2_) in the headspace of the vials as well as ethanol and the carboxylic acids propionic acid, acetic acid, lactic acid, formic acid, butyric acid, isobutyric acid, valeric acid and isovaleric acid in the medium residues were analyzed using high performance liquid chromatography resp. gas chromatography as described by (Hahnke et al., 2016).

## Results and discussion

### General features of the genome of *Anaeropeptidivorans aminofermentans* M3/9^T^

**Figure 1:**
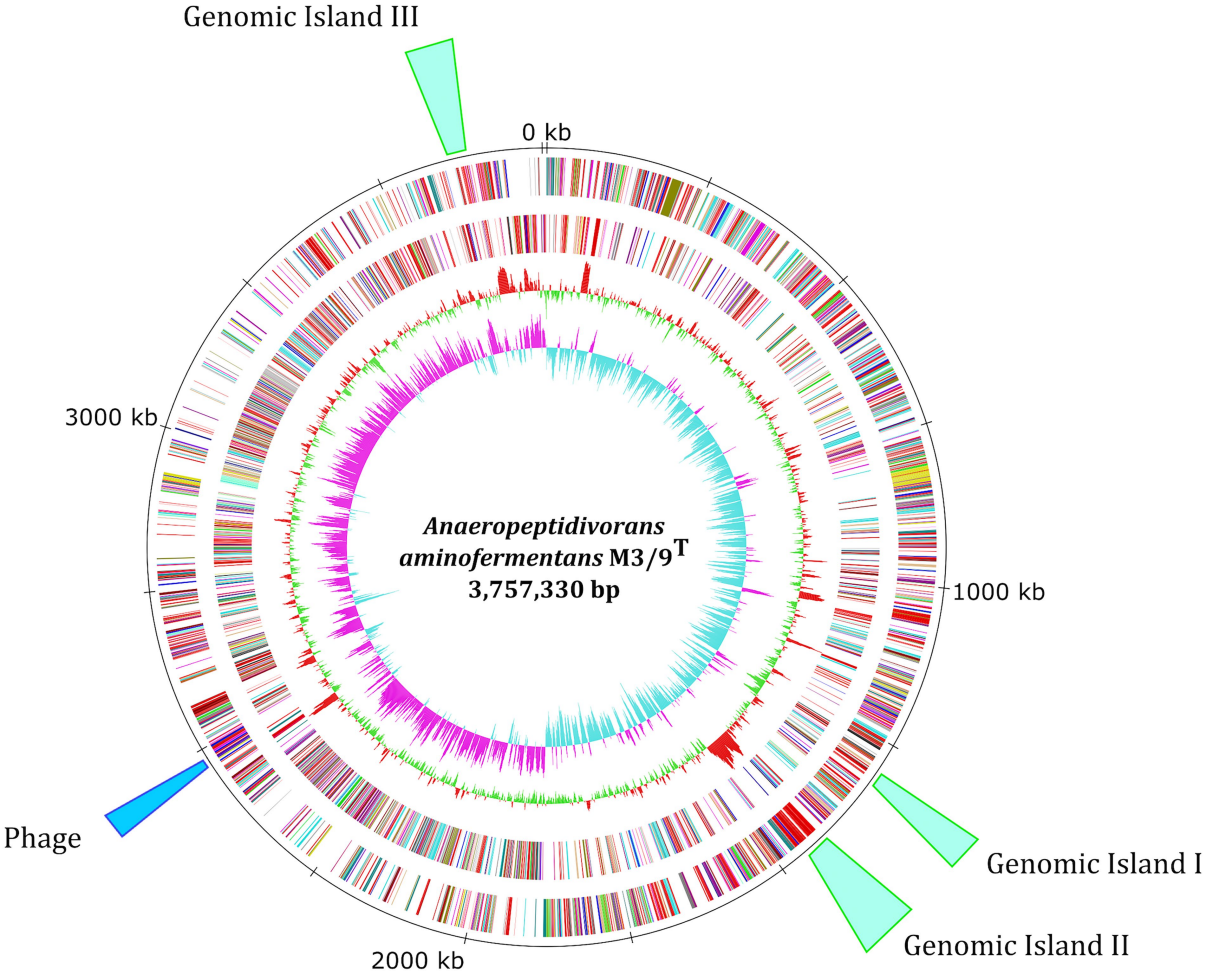
Circular representation of the *A. aminofermentans* M3/9^T^ chromosome. From the inner to the outer circle: Circle 1, GC skew; Circle 2, GC content; Circles 3 and 4, predicted protein-coding sequences (CDS) transcribed clockwise (outer part) or anticlockwise (inner part). The CDSs are colored according to the assigned COG classes. Circle 5, genomic position in kb. Circle 6, predicted prophage cluster and three genomic island regions in the *A. aminofermentans* M3/9^T^ genome. The replication initiation gene *dna*A was chosen as the first gene of the chromosome.

To deduce the role of a new and unknown member in biogas-producing microbial communities, the *A. aminofermentans* M3/9^T^ genome was sequenced, finished and analyzed in detail. General features of the M3/9^T^ genome are summarized in Table 1 and the corresponding genome plot is shown (Figure 1). Genome assembly with PacBio long reads followed by Illumina short-read polishing resulted in one circular chromosome with a size of 3,757,330 bp and a GC-content of 38.45%. Genome annotation of *A. aminofermentans* M3/9^T^ resulted in the prediction of 3,110 coding sequences, 55 tRNA genes, and five *rrn* operons. In the genome of *A. aminofermentans* M3/9^T^, three genomic island regions (Figure 1) and putative phage genes were identified with IslandViewer 4 and PHASTER. However, one incomplete prophage cluster (2,530,530 bp - 2,561,909 bp) was also predicted in the genome. In addition, strain M3/9^T^ harbors one CRISPR it system that may be involved in preventing the invasion of phages and mobile genetic elements. Based on the CARD analysis, *A. aminofermentans* M3/9^T^ also may possess a putative vancomycin resistance cluster consisting of seven genes (*van*X, *van*B, *van*H, *van*W, *van*Y, *van*S and *van*R).

**Table 1:** General features of the *Anaeropeptidivorans aminofermentans* M3/9^T^ genome.

Feature	Chromosome
Genome size (bp)	3,757,330
GC content (%)	38,45
Protein coding genes	3,110
*rrn* operons	5
tRNA genes	55

### Phylogenetic affiliation of *Anaeropeptidivorans aminofermentans* M3/9^T^ with other genome sequenced prokaryotes

Based on 16S rRNA gene sequence and on genome sequence comparisons, i.e., average amino acid identities (AAI) and the percentage of conserved proteins (POCP), *A. aminofermentans* M3/9^T^ was recently classified as a new species of a novel genus within the family *Lachnospiraceae* (phylum *Bacillota,* syn*. Firmicutes* (Köller et al., 2022). Phylogenetic analyses revealed a close relationship of strain M3/9^T^ to the *Anaerotignum* sub-cluster (Köller et al., 2022).

**Figure 2:**
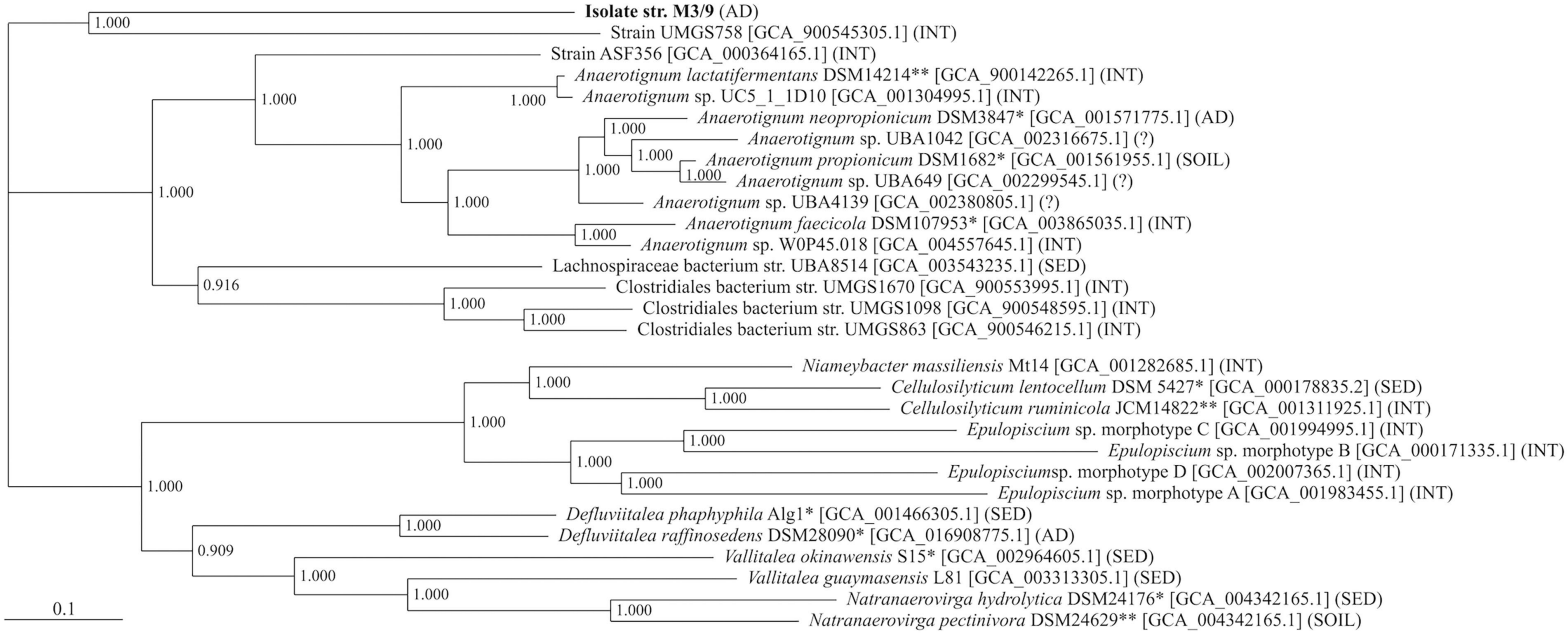
Phylogenetic affiliation of *A. aminofermentans* M3/9^T^. The phylogenetic tree is based on all core genes of the selected strains from the family *Lachnospiraceae* as determined by means of the comparative genomics tool EDGAR. The bar below the phylogenetic tree represents the scale of sequence divergence. Sampling site: AD, anaerobic digester; INT, intestinal microbiome; SED, marine or freshwater sediment; SOIL, soil;?, unknown sampling site. *, species type strain; **, genus type strain.

Further, the genome sequence of stain M3/9^T^ was compared to related, publicly available genomes of the family *Lachnospiraceae*, and a genome-based phylogenetic tree was constructed within the comparative genomics tool EDGAR. The phylogenetic tree supported the close affiliation of strain M3/9^T^ with the genus *Anaerotignum* (Figure 2), and showed a rather distant relationship with other genera, e.g., *Cellulosilyticum*, *Epulopiscium*, *Niameybacter*, *Defluviitalea*, *Vallitalea*, and *Natranaerovirga*, comprising (obligate) anaerobic, mesophilic or thermophilic species.

The genus *Anaerotignum* comprises chemoorganotrophic species utilizing amino acids and producing short-chain volatile fatty acids (VFA), mainly acetate, propionate, and butyrate, as end-products (Ueki et al., 2017; Choi et al., 2019). Glucose is not or only weakly fermented; the capacity to utilize xylose is limited to *A. neopropionicum* and *A. lactatifermentans* (Ueki et al., 2017). In addition to VFA, most species produce molecular H_2_ as end-product of fermentation. Type strains for *Anaerotignum* species were isolated from anaerobic digesters, but also from black mud samples and human feces (Ueki et al., 2017; Choi et al., 2019).

Beside type strains, several MAGs showing some similarities to the M3/9^T^ genome are deposited in public databases (Figure 2). Most closely related to strain M3/9^T^ was MAG UMGS758 (accession number: GCA900545305), a genome sequence derived from large-scale metagenome sequencing of 11,850 human gut microbiomes (Almeida et al., 2019). However, its phylogenetic distance to strain M3/9^T^ is still quite high. The occurrence of MAG UMGS758 as part of intestinal microbiota is indicating a lifestyle adapted to biomass degradation at low-oxygen resp. anaerobic conditions, which is probably similar to the lifestyle of strain M3/9^T^ and the other genera of this *Lachnospiraceae* cluster.

In summary, genome wide similarities with members of the genus *Anaerotignum* lead to the assumption that strain M3/9^T^ is a functional member of anaerobic biomass degrading microbiomes acting in proteolysis, primary carbohydrate breakdown and/or secondary fermentation of carbohydrates and/or amino acids.

### Genes encoding enzymes of the central fermentation metabolism

*Anaeropeptidivorans aminofermentans* M3/9^T^ encodes complete KEGG modules for glycolysis (Embden-Meyerhof pathway, M00001), pyruvate oxidation (M00307), and PRPP (phosphoribosyl pyrophosphate) biosynthesis (M00005) allowing metabolism of glucose and other monosaccharides. However, the carbohydrate and sugar metabolism of the strain is limited as revealed by its carbohydrate-active enzymes profile (CAZymes). The M3/9^T^ genome only encodes one predicted glycoside hydrolase (GH18 family), two multiple sugar transport systems and one simple sugar transport system.

**Figure 3:**
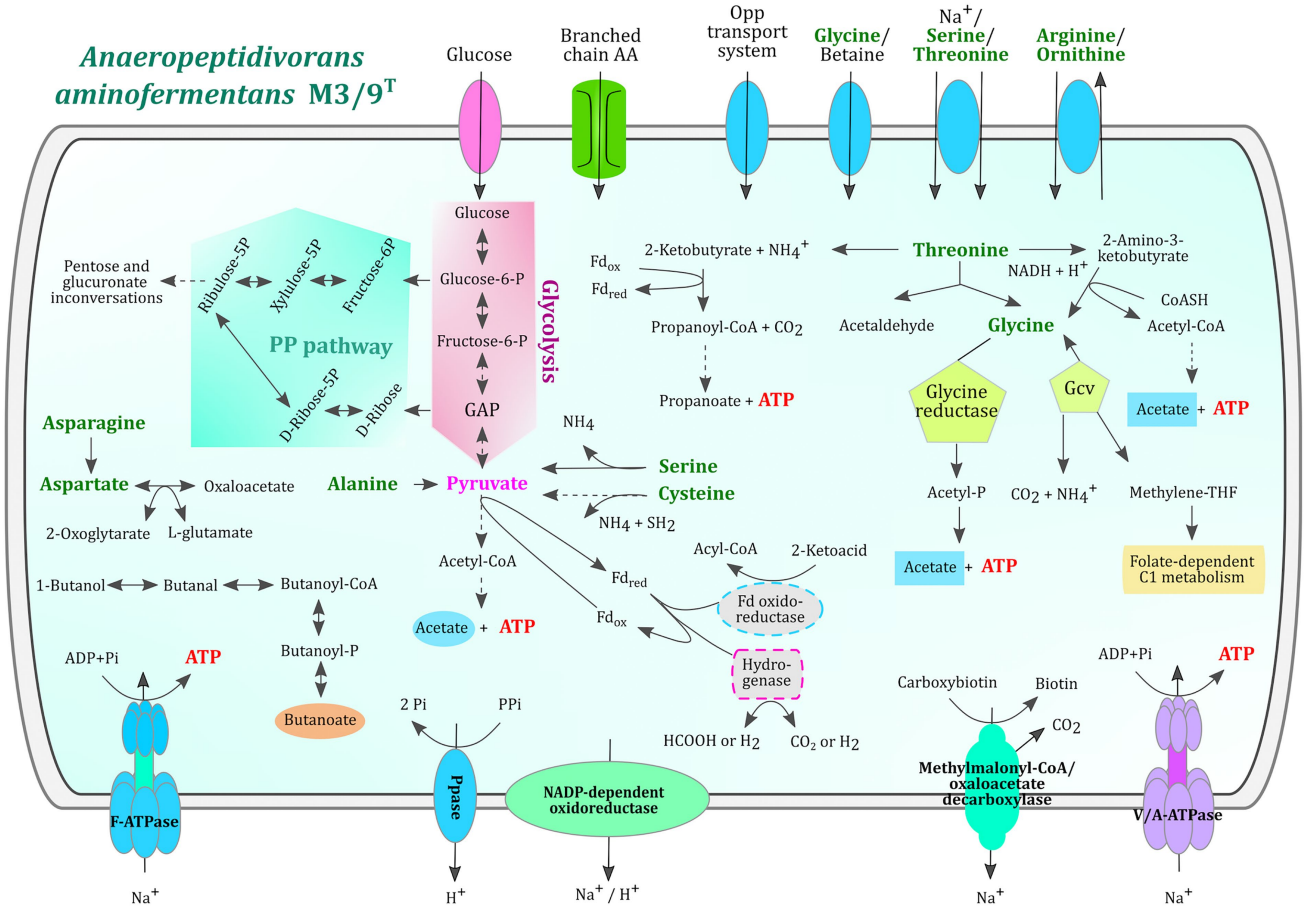
Metabolic reconstruction of relevant metabolic features and amino acid utilization in *A. aminofermentans* M3/9^T^. The metabolic reconstruction was performed with KEGG pathway maps represented in GenDB. The glycolysis and the pentose phosphate pathways are highlighted in pink and green, respectively. Amino acids proposed to be utilized by *A. aminofermentans* M3/9^T^ are written in green and corresponding transport systems are displayed in blue ovals. Metabolic pathways shown in the figure are completely encoded in the genome.

*Anaeropeptidivorans aminofermentans* M3/9^T^ is also predicted to degrade proteins, oligo- and dipeptides since proteinases, oligopeptidases and dipeptidases are encoded in its genome (see Supplementary Tables S2, S3). The comparison of M3/9^T^ genes with entries within the MEROPS database representing structure-based classification of the peptidases revealed in total 69 hits. The majority of them were assigned to the families M20 (encoding for exopeptidases: carboxypeptidases, dipeptidases and a specialized aminopeptidase), M24 (type IV prepilin peptidase 1), M42 (virus-type papain-like peptidase), S11 (serine-type D-Ala-D-Ala carboxypeptidases) and various other families representing proteolytic enzymes (Rawlings et al., 2018; Kieliszek et al., 2021; Solanki et al., 2021; Christensen et al., 2022). In accordance with this, several oligopeptide transporter gene clusters (e.g., *opp*ABCDF) and genes encoding transporters for the amino acids leucine, isoleucine, valine (*liv*FGMHK), arginine, lysine, histidine, glycine, proline, tryptophan, tyrosine and polar amino acids were identified in the M3/9^T^ genome. Amino acids released from peptide hydrolysis are most likely further metabolized since strain M3/9^T^ features a very versatile amino acid metabolism (Figure 3). For example, KEGG modules for valine, leucine, isoleucine, lysine, ornithine, arginine, proline, histidine, cysteine, and methionine metabolism are complete.

Concerning the threonine metabolism, the M3/9^T^ genome encodes three key enzymes, namely threonine ammonia-lyase (EC: 4.3.1.19), L-threonine-3-dehydrogenase (EC: 1.1.1.103), and L-threonine aldolase (EC: 4.1.2.48). The product of the reaction catalyzed by the first enzyme, 2-ketobutyrate, can be metabolized to propionate *via* propionyl-CoA yielding ATP. All necessary enzymes of this pathway were predicted in the M3/9^T^ genome. Secondly, threonine can be oxidized to 2-amino-3-ketobutyrate that subsequently is converted to glycine and acetyl-CoA (glycine C-acetyltransferase, EC: 2.3.1.29). ATP generation occurs within the phosphate-acetyltransferase/acetate kinase module (KEGG M00579) releasing acetate. Finally, threonine can be cleaved into acetaldehyde and glycine entering the glycine reductase/glycine-cleavage-system (Gcv) pathway. The Gcv-system (EC: 1.4.4.2, EC: 2.1.2.10 and EC: 1.8.1.4) converts glycine to carbon dioxide, ammonium and methylene-tetrahydrofolate whereas glycine reductase (EC: 1.21.4.2) releases acetyl-phosphate that subsequently yields acetate and ATP through the activity of acetate kinase (EC: 2.7.2.1). Glycine reductase is regarded as the key enzyme of the Stickland reaction (Fonknechten et al., 2010). The enzyme cysteine-S-conjugate β-lyase (EC: 4.4.1.13) of strain M3/9^T^ may be involved in conversion of cysteine to pyruvate that is further metabolized in the pyruvate module (KEGG M00307) for ATP generation. Likewise, the enzyme L-serine ammonia-lyase (EC: 4.3.1.17) catalyzes the reaction of serine to pyruvate. Serine may also be converted to glycine by the enzyme glycine hydroxymethyltransferase (EC: 2.1.2.1). L-alanine is converted to pyruvate and glycine by the alanine-glyoxylate transaminase (EC: 2.6.1.44) and also the pathway from L-aspartate to pyruvate is present in strain M3/9^T^ involving the enzymes L-aspartate oxidase (EC: 1.4.3.16), L-aspartate transaminase (EC: 2.6.1.1), phosphoenolpyruvate carboxykinase (EC: 4.1.1.32) and pyruvate kinase (EC: 2.7.1.40) or oxaloacetate decarboxylase (Na^+^-extruding; EC: 7.2.4.2). L-asparagine can be converted to L-aspartate by aspartate-ammonia ligase (EC: 6.3.1.1). Accordingly, complete pathways for fermentation of the amino acids threonine, glycine, cysteine, serine, alanine, aspartate and asparagine were identified in strain M3/9^T^. In contrast to *Clostridium sticklandii* (Fonknechten et al., 2010), strain M3/9^T^ does not encode all key enzymes for the degradation of arginine and proline.

Concerning the butanoate pathway, the M3/9^T^ genome encodes all enzymes for the conversion of butanol to butanoate, namely butanol dehydrogenase (EC: 1.1.1.-), acetaldehyde dehydrogenase (EC: 1.2.1.10), phosphate butyryltransferase (EC: 2.3.1.19) and butyrate kinase (EC: 2.7.2.7). For the latter enzyme, three copies of the *buk* gene were identified in the M3/9^T^ genome. Since the enzyme crotonyl-CoA reductase (butyryl-CoA dehydrogenase, EC: 1.3.1.86) is not encoded in the M3/9^T^ genome, the metabolites lysine, acetyl-CoA, 2-oxoglutarate, succinate and 4-aminobutyrate probably cannot be converted to butyrate. Butyrate synthesis pathways involving the latter metabolites were depicted in a previous publication (Vital et al., 2014).

Enzymes involved in the propanoate pathway were described above in the context of threonine fermentation pathways. In summary, strain M3/9^T^ may synthesize ATP by substrate-level phosphorylation from acetyl-phosphate, propanoyl-phosphate, butyryl-phosphate, 1,3-diphosphoglycerate, phosphoenol-pyruvate and 10-formyl-tetrahydrofolate facilitated by formate-tetrahydrofolate ligase (EC: 6.3.4.3).

The experimental analysis of fermentation end-products carried out in parallel to the genome analysis revealed that strain M3/9^T^ produced large amounts of acetic acid, smaller amounts of ethanol, formic acid, isovaleric acid, lactic acid and isobutyric acid as well as carbon dioxide. These metabolites can be explained by the above reconstructed metabolism. The detection of propionic acid, butyric acid, valeric acid and H_2_ failed under applied cultivation conditions.

*Anaeropeptidivorans aminofermentans* M3/9^T^ possesses F- and V/A-type ATPases for the exploitation of a Na^+^/H^+^ gradient driving ATP synthesis. Na^+^/H^+^ gradients probably are build up by the activity of an Na^+^-extruding oxaloacetate or methylmalonyl-CoA decarboxylase (Lietzan and St. Maurice, 2014) and a proton-exporting inorganic pyrophosphatase. In contrast to *C. sticklandii*, strain M3/9^T^ does not possess an Rnf-complex (Ferredoxin-NAD^+^ oxidoreductase, Na^+^-transporting**)** facilitating the export of Na^+^ and H^+^ ions.

Like *C. sticklandii*, strain M3/9^T^ also harbors a hydrogenase gene region consisting of eight genes. Two of them encode the small and large subunit of a periplasmic [NiFe] group 1a hydrogenase whereas the products of the remaining genes have accessory functions (maturation, formation/expression). Reference group 1a hydrogenases are described as uptake hydrogenases (hydrogenotrophic) facilitating oxidation of H_2_ for energy recovery [HydDB; (Søndergaard et al., 2016)]. A second putative hydrogenase gene region is composed of four genes encoding a NADP-dependent oxidoreductase (subunit E), a 2Fe-2S ferredoxin-domain-containing protein, a 4Fe-4S dicluster domain-containing protein (also annotated as NADH-quinone oxidoreductase, subunit NuoF) and a 2Fe-2S iron–sulfur-cluster-domain-containing protein (also annotated as small subunit of a [FeFe] group A hydrogenase). The complex may function in a redox process involving NADH, a quinone and probably H_2_. Whether the catalyzed redox reaction is connected to proton (or Na^+^) translocation remains speculative.

### Comparative genome analyses of *Anaeropeptidivorans aminofermentans* M3/9^T^ with closely related strains from the genus *Anaerotignum*

**Figure 4:**
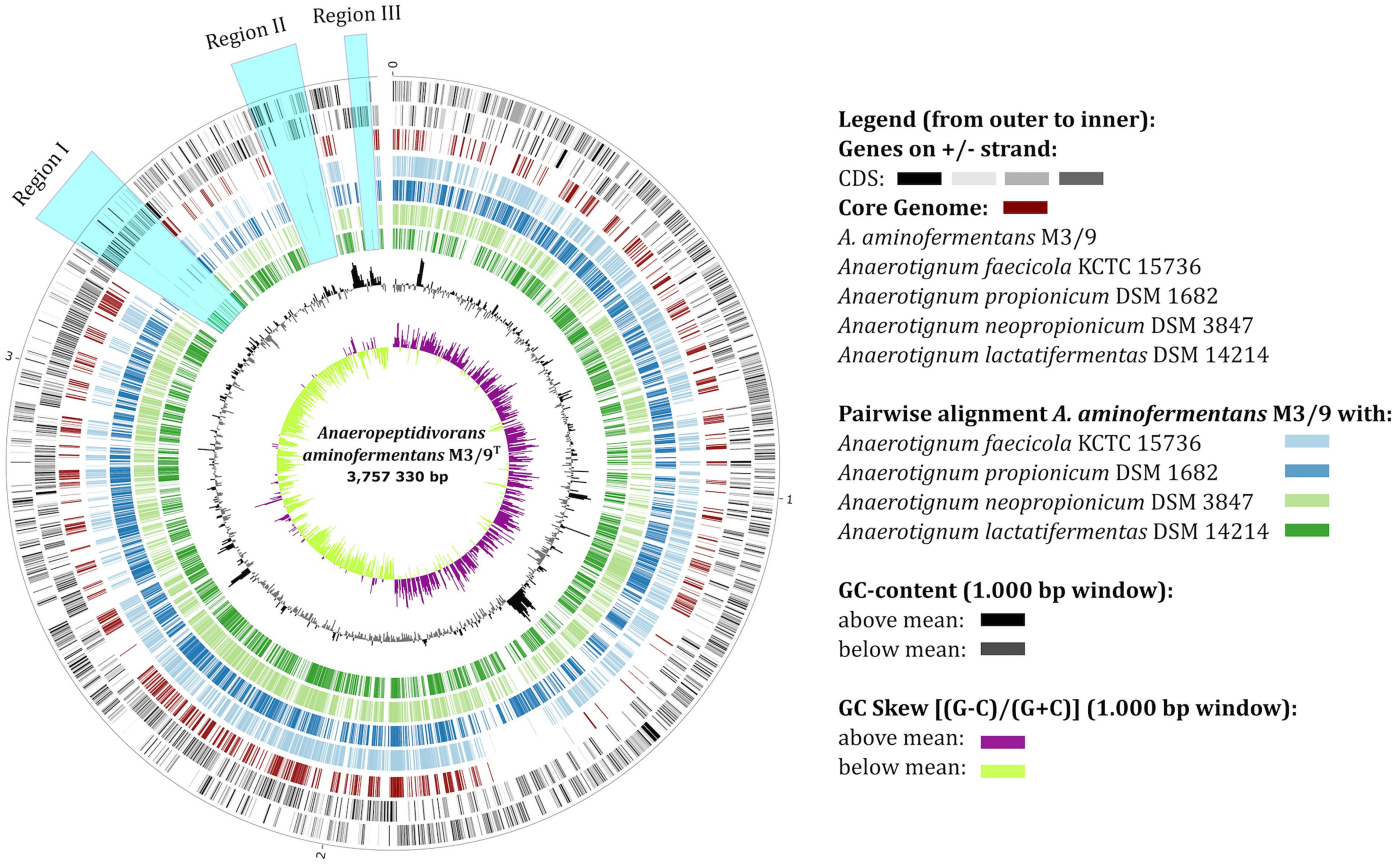
Multi-genome circular plot visualizing the orthologous genes in the genomes of *A. aminofermentans* M3/9^T^ and closely related *Anaerotignum* strains. The black outer rings of the circular plot represent the genes of the *A. aminofermentans* M3/9^T^ genome. The further rings of the circular plot show the core genome (red) as well as the orthologs of each individual *Anaerotignum* genome in comparison to the reference.

The phylogenetic allocation of *A. aminofermentans* M3/9^T^ based on core gene analyses (Figure 2) as well as on classification of its 16S rRNA gene sequence (Supplementary Figure S2) indicated a close relationship to representatives of the genus *Anaerotignum*. To understand the genomic diversity between the biogas isolate M3/9^T^ and validly described *Anaerotignum* type strains for which genome sequences are available, namely *Anaerotignum propionicum* DSM 1682^T^ (Cardon and Barker, 1946), *A. neopropionicum* DSM 3847^T^ (Tholozan et al., 1992), *A. lactatifermentans* DSM 14214^T^ (van der Wielen et al., 2002) and *Anaerotignum faecicola* KCTC 15736^T^ (Choi et al., 2019), genome sequence comparisons were performed applying the tool EDGAR (Dieckmann et al., 2021). The organization of the M3/9^T^ chromosome revealed three large unique regions (regions I-III) compared to the reference genomes of the genus *Anaerotignum* (Figure 4). The region I is 131,739 bp long and contains i.a. genes encoding the uptake system Liv for L-leucine, L-valine, and L-isoleucine transport, the oligopeptide transport system Ami and the glycine cleavage system Gcv (EC: 1.4.4.2) catalysing the degradation of glycine. The region II is 90,515 bp long and harbors the ABC transporter complex MetNIQ responsible for methionine import, the ABC transporter complex Fhu involved in iron (3^+^)-hydroxamate import and a complete class 2, type VI CRISPR-Cas system (start/end positions at 3,579,842–3,590,818 bp) as deduced by applying the CRISPRFinder tool (Grissa et al., 2007). Finally, region III (30,516 bp) contains genes encoding the uncharacterized ABC transporter permease protein Yuf. Thus, it is conceivable that the chromosome of the biogas plant isolate *A. aminofermentans* M3/9^T^ has been shaped to achieve adaptation to its specific habitat.

**Figure 5:**
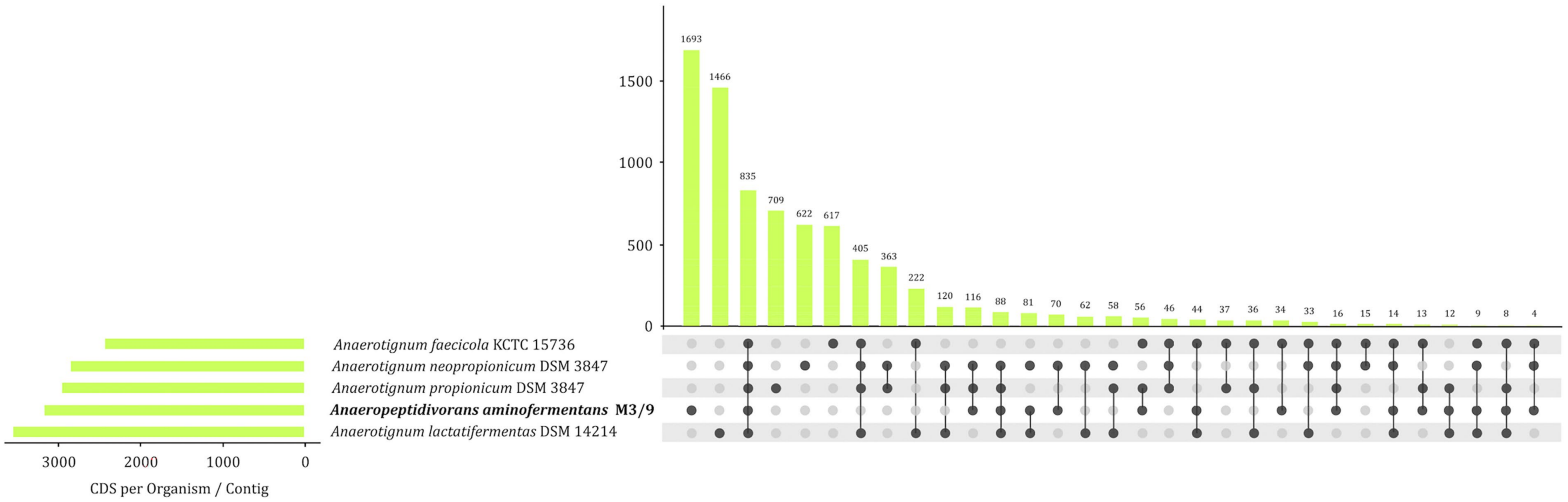
UpSet diagram illustrating the core genome and common genes among *A. aminofermentans* M3/9^T^ and closely related *Anaerotignum* species based on complete genome sequences of the type strains.

EDGAR was also applied to calculate the set of species-specific and shared protein-coding genes of strain M3/9^T^ and members of the genus *Anaerotignum* (Figure 5). This analysis only revealed 835 homologous genes shared by the analyzed bacterial genomes indicating large genomic diversity in the strains analyzed. Core genes encode the complete glycolysis pathway and proteins/enzymes involved in sugar uptake and fermentation. In contrast, the biogas plant isolate *A. aminofermentans* M3/9^T^ harbors 1,693 genes for proteins with no homologous counterparts in the *Anaerotignum* genomes. These strain-specific genes encode 410 hypothetical proteins, phage related proteins, different peptidases, the Xaa-Pro aminopeptidase rXpmA, a phosphotransferase system (PTS) for glucose, mannose, fructose and sorbose uptake, but also proteins involved in extracellular solute binding and import, sporulation and flagellar biosynthesis. Unique genes encoded in the *A. aminofermentans* M3/9^T^ genome represent those genetic determinants that may specify characteristic features of this species.

### Occurrence of *Anaeropeptidivorans aminofermentans* M3/9^T^ relatives in microbial communities as deduced from publicly available metagenome data

Discovery of a new species often leads to the question which niche this new organism occupies in the environment. What role does it play in the microbiome and how abundant are members of this new taxon in the community? To determine the occurrence of *A. aminofermentans* M3/9^T^ and its closest relatives, namely *A. faecicola* DSM 107953^T^, *A. lactatifermentans* DSM 14214^T^, *A. neopropionicum* DSM 3847^T^ and *A. propionicum* DSM 1682^T^, in the environment, 477,981 sequence read archive (SRA) accessions representing different metagenome data sets from various habitats were screened applying the tool Mash Screen (Ondov et al., 2019). Due to the scaling capabilities of the de.NBI Cloud and a fast connection between the S3 cloud storage system and the virtual machines, 437 Terabyte of sequence data could be screened in a short period of time. Since Mash Screen just estimates the containment of a genome in a metagenome, more accurate metagenome fragment mappings were additionally performed applying the BWA read alignment software package.

Members closely related to the above-mentioned bacteria were detected by Mash in 265,282 out of 477,981 SRA data sets. By applying stricter search settings, namely at least 700 shared kmer hashes, 509 hits in total remain. Obtained metagenome data sets were then aligned against five genomes using BWA. Final matches are only reported if at least 95% of the genome is covered by at least one read per base. The trimmed mean coverage of the filtered matches was depicted in (Supplementary Figure S2).

**Figure 6:**
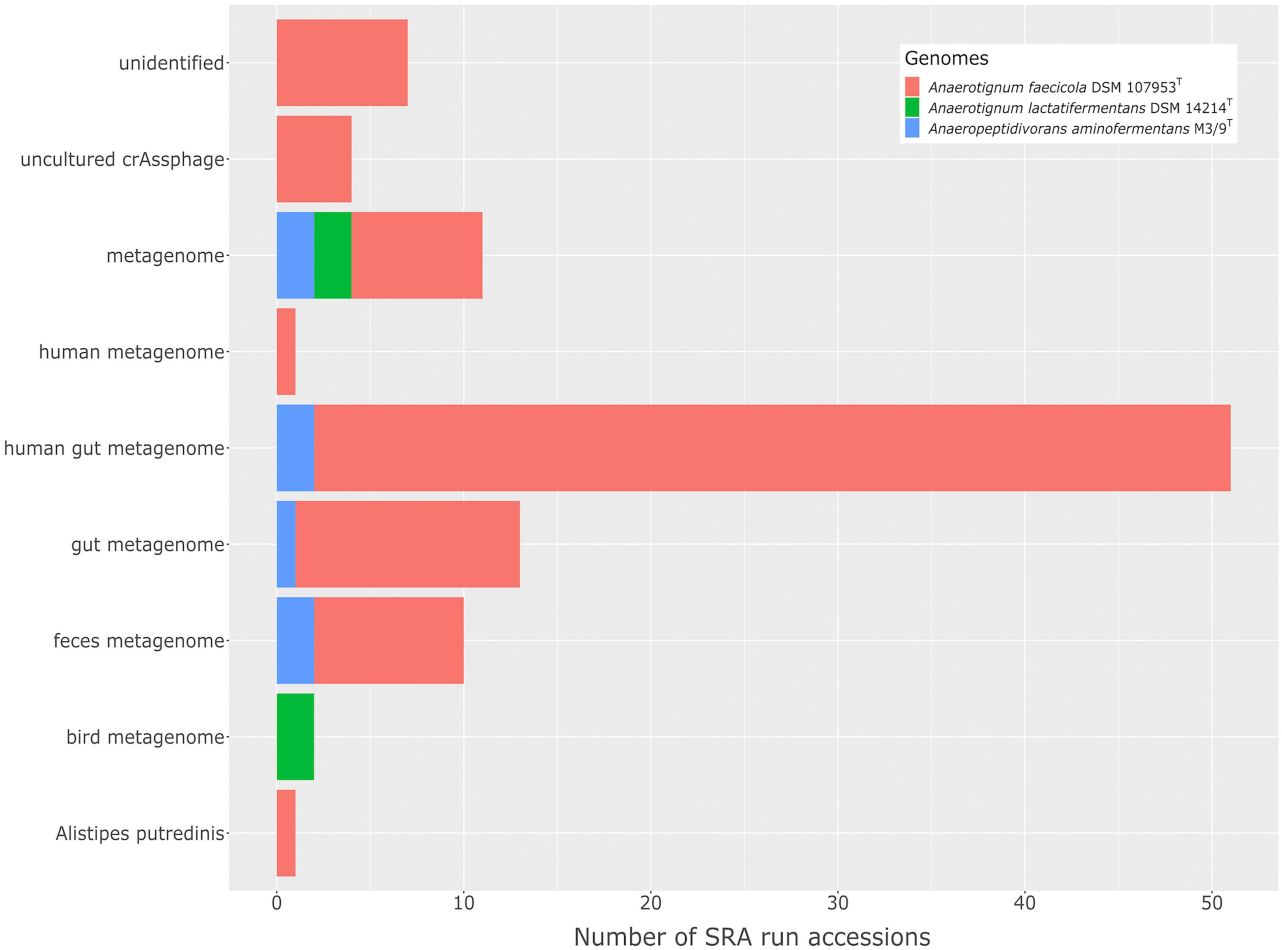
The distribution of *A. aminofermentans* M3/9^T^ and closely related *Anaerotignum* species in different environments as deduced from metagenome fragment mappings.

Metagenome samples representing human gut microbiomes featured the highest abundance of *A*. *aminofermentans* relatives, belonging to the genus *Anaerotignum*, ranging between 0.46 and 2.34% of the entire microbiome (Figure 6). Microbiome members closely related to A. *faecicola* were found to be widespread (89 out of 100 SRA data sets) and were among others frequently identified in feces microbial communities. *A. lactatifermentans* was occasionally detected in bird and chicken gut microbiomes. Since also the type strain *A. faecicola* DSM107953^T^ was isolated from the feces of a healthy Korean (Choi et al., 2019) and *A. lactatifermentans* DSM14214^T^ originated from the caeca of a 31-day-old chicken (van der Wielen et al., 2002), obtained results are in line with previous findings. Thus, *Anaerotignum* species show an adaptation to corresponding habitats such as the human gut and bird microbiome, respectively.

**Figure 7:**
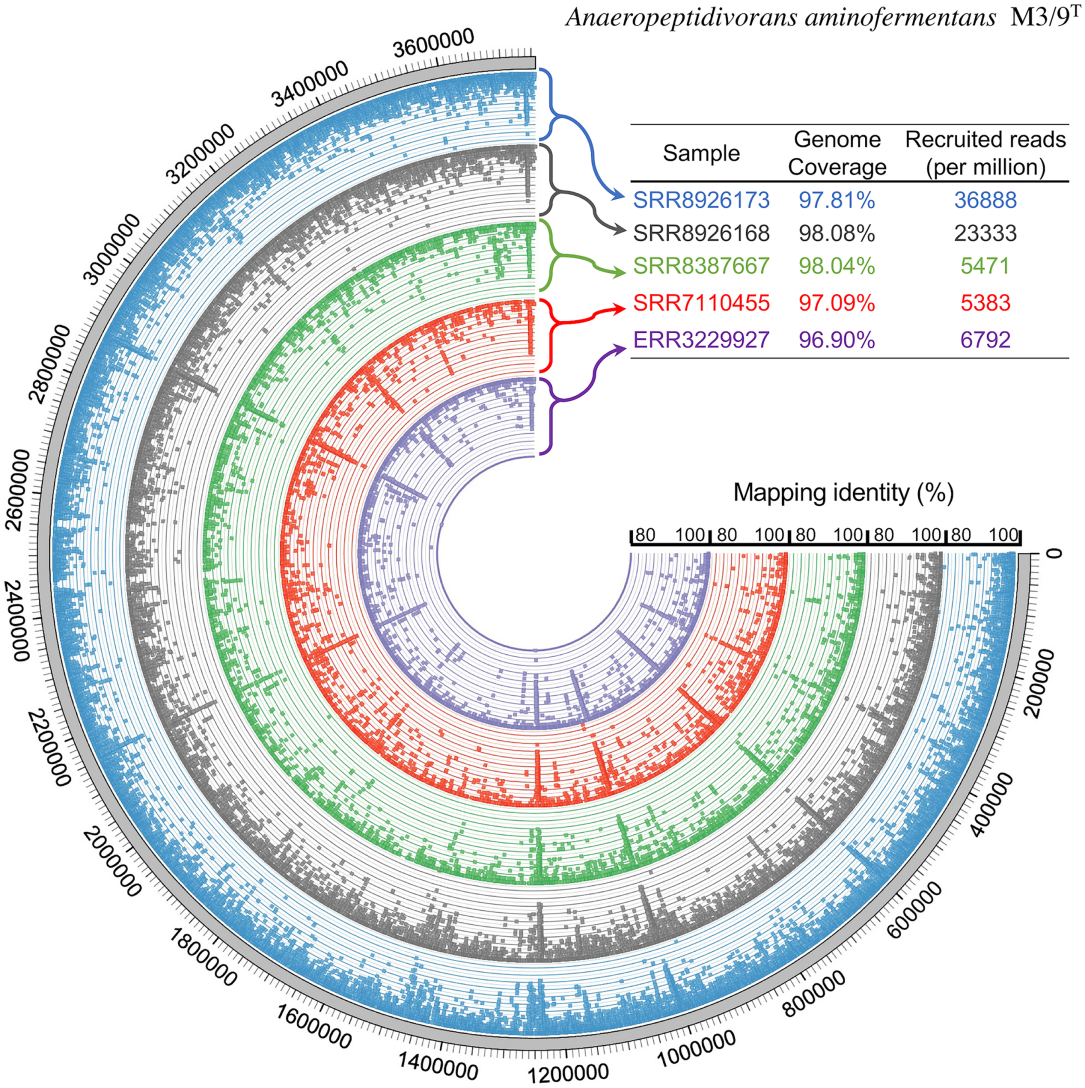
Fragment recruitment of publicly available metagenome sequences derived from the human gut and goat fecal pellet samples and mapped onto the M3/9^T^ genome sequence. Five selected samples showing an alignment of reads to M3/9^T^ genome which have a coverage of at least 95% and a mapping identity of at least 95%. The outer ring represents the genome in base pairs, while the remaining colored rings show the coverage of the corresponding metagenome sequences mapped onto the genome sequence. Sample origin: SRR8926173 and SRR8926168, human gut metagenome originating from intensive care unit patient; SRR8387667, human gut metagenome of cancer patient; SRR7110455, metagenome representing the goat fecal pellet microbiome; ERR3229927, gut metagenome.

*Anaeropeptidivorans aminofermentans* was detected in seven out of 509 SRA data sets analyzed (see Figure 6), showing genome coverages being at least 95%. For analyzing the occurrence of the species *A. aminofermentans*, we restricted found matches not only by the genome coverage but also by a mapping identity of at least 95% which resulted in five matches. *Anaeropeptidivorans* and its relatives were found in metagenomes originating from the human gut as well as goat fecal pellet samples. Results of these metagenome fragment mapping analyses were visualized in a circos alignment plot (Figure 7). Approximately, 0.5 to 4.9% of all metagenome reads were mapped on the M3/9^T^ genome. In addition to the human gut, *A. aminofermentans* was also found to be abundant in the microbiome representing goat fecal pellets enriched with Alfalfa or Reed Canary Grass, Isla Vista (accession number: SRR7110455). Approximately, 0.6% of the goat pellet metagenomic reads featured a match with a high mapping identity against the reference. This result indicated that *A. aminofermentans* is adapted to the mammalian intestine, represented, e.g., by goat feces, which sometimes even is used as a substrate for biogas production.

## Conclusion

Understanding the microbial ecology of AD, and how far AD microbiomes are functionally similar to biogas microbiomes, requires identification and isolation of active and important microorganisms and linking their genome information to their functional roles. Furthermore, future success on good data interpretation depends heavily on developing and improving databases for collating microorganisms and enzymes responsible for the biodegradation of biomass. Genomic technologies and integrated omics analyses are tools with enormous potential for increasing our understanding of microorganisms. It has become the standard method for predicting the metabolic potential of microorganisms and has clear advantages compared to other approaches in characterizing and subtyping of strains.

In this study, the genome sequence of *A. aminofermentans* M3/9^T^, recently classified as a new species within the new genus *Anaeropeptidivorans* (Köller et al., 2022), isolated from a mesophilic laboratory-scale biogas reactor, was analyzed in detail including genome-based metabolic reconstruction. A comparative analysis of strain M3/9^T^ with closely related *Anaerotignum* species contributed to a better understanding of the genetic basis supporting the growth and competitiveness of the isolate in the biogas reactor environment. Applying cloud computing for large-scale genomics and metagenomics analysis provided a better understanding of the distribution of the species *A. aminofermentans* in the environment, with this demonstrating a clear advantage of this technique for genomics research.

Insights into the strain’s genetic repertoire and metabolic potential revealed that the M3/9^T^ genome encodes limited carbohydrate and sugar metabolism enzymes as derived from its carbohydrate-active enzyme profile. The isolate was predicted to only utilize the monosaccharide glucose in terms of energy production and storage, even though, in cultivation experiments the strain did not show utilization of glucose using the API 20 A identification assay (bioMérieux) (Köller et al., 2022). However, strain M3/9^T^ possesses genetic determinants needed to degrade proteins, oligo- and dipeptides. It is also able to utilize amino acids as a carbon source since the corresponding genes were identified in the M3/9^T^ genome. This is in accordance with the fact that strain M3/9^T^ grew in medium containing complex proteinaceous substrates (Köller et al., 2022). Furthermore, acetic acid, ethanol, formic acid, isovaleric acid, lactic acid, isobutyric acid, and CO_2_, representing fermentation end-products, have also been experimentally proven. This is again in accordance with results obtained from the metabolic pathway reconstruction, which shed light on the function of *A. aminofermentans* M3/9^T^ in the AD process associated with acidogenesis.

A fragment recruitment approach exploiting almost half a million of publicly available metagenome data sets provided insights into the distribution of this species in the environment. Obtained results indicated that *A. aminofermentans* and its closely related members from the genus *Anaerotignum* are widespread in AD systems. More detailed, *A. aminofermentans* was found particularly abundant in the human gut, but also in the microbiome of the mammalian intestine. *A. aminofermentans* probably enters the biogas reactor *via* mammalian excrement, which is used as a substrate for biogas production.

The availability of the complete genome sequence of the strain *A. aminofermentans* M3/9^T^ will provide the opportunity to study differences in genome structures and contents among *Anaeropeptidivorans* and closely related *Anaerotignum* species with the aim to identify potential adaptive features regarding their specific habitats.

## Data availability statement

The datasets presented in this study can be found in online repositories. The names of the repository/repositories and accession number(s) can be found at: https://www.ncbi.nlm.nih.gov/genbank/, PRJEB52185.

## Author contributions

IM, AP, AnS, and MK conceptualized and proposed the idea of the manuscript. BB and CS performed the sequencing and genome assembly. SH cultivated the strain and conducted DNA extraction. IM, PB, DW, LH, and AlS provided bioinformatics analysis. IM, AnS, MK, and SH wrote the first draft of the manuscript. All authors contributed to the article and approved the submitted version.

## Funding

This research was funded in the context of the joint project BIOGAS-CORE granted by the German Federal Ministry of Food and Agriculture (grant no. 22017111) and supported by the German Fachagentur Nachwachsende Rohstoffe. AnS and AlS acknowledge funding by the European Union’s Horizon 2020 Research and Innovation Program under the grant agreement number 818431 (SIMBA, Sustainable Innovation of Microbiome Applications in Food Systems). This work was also supported by the BMBF-funded de.NBI Cloud within the German Network for Bioinformatics Infrastructure (de.NBI) (031A532B, 031A533A, 031A533B, 031A534A, 031A535A, 031A537A, 031A537B, 031A537C, 031A537D, and 031A538A).

## Conflict of interest

IM, DW, PB and AS were employed by company Forschungszentrum Jülich GmbH. CS and BB were employed by company Leibniz Institute DSMZ-German Collection of Microorganisms and Cell Cultures GmbH.

The remaining authors declare that the research was conducted in the absence of any commercial or financial relationships that could be construed as a potential conflict of interest.

## Publisher’s note

All claims expressed in this article are solely those of the authors and do not necessarily represent those of their affiliated organizations, or those of the publisher, the editors and the reviewers. Any product that may be evaluated in this article, or claim that may be made by its manufacturer, is not guaranteed or endorsed by the publisher.

## Footnotes

^1^ https://www.genome.jp/kegg/

^2^ https://www.denbi.de/cloud

^3^ https://github.com/wwood/CoverM

^4^ https://github.com/BiBiServ/bibigrid

## References

[ref1] Almeida A. Mitchell A. L. Boland M. Forster S. C. Gloor G. B. Tarkowska A.. (2019). A new genomic blueprint of the human gut microbiota. Nature 568, 499–504. doi: 10.1038/s41586-019-0965-1, PMID: 30745586 PMC6784870

[ref2] Arndt D. Grant J. R. Marcu A. Sajed T. Pon A. Liang Y.. (2016). PHASTER: a better, faster version of the PHAST phage search tool. Nucleic Acids Res. 44, W16–W21. doi: 10.1093/nar/gkw387, PMID: 27141966 PMC4987931

[ref3] Beck M. H. Poehlein A. Bengelsdorf F. R. Schiel-Bengelsdorf B. Daniel R. Dürre P. (2016). Draft genome sequence of the strict anaerobe clostridium neopropionicum X4 (DSM 3847T). Genome Announc. 4, e00209–e00216. doi: 10.1128/genomeA.00209-16, PMID: 27081124 PMC4832152

[ref4] Bertelli C. Laird M. R. Williams K. P. Lau B. Y. Hoad G. Winsor G.. (2017). IslandViewer 4: expanded prediction of genomic islands for larger-scale datasets. Nucleic Acids Res. 45, W30–W35. doi: 10.1093/nar/gkx343, PMID: 28472413 PMC5570257

[ref5] Cai S. Dong X. (2010). Cellulosilyticum ruminicola gen. Nov., sp. nov., isolated from the rumen of yak, and reclassification of clostridium lentocellum as cellulosilyticum lentocellum comb. nov. Int. J. Syst. Evol. Microbiol. 60, 845–849. doi: 10.1099/ijs.0.014712-0, PMID: 19661493

[ref6] Campanaro S. Treu L. Rodriguez-R L. M. Kovalovszki A. Ziels R. M. Maus I.. (2020). New insights from the biogas microbiome by comprehensive genome-resolved metagenomics of nearly 1600 species originating from multiple anaerobic digesters. Biotechnol. Biofuels 13:25. doi: 10.1186/s13068-020-01679-y, PMID: 32123542 PMC7038595

[ref7] Cardon B. P. Barker H. A. (1946). Two new amino-acid-fermenting bacteria, clostridium propionicum and *Diplococcus glycinophilus*. J. Bacteriol. 52, 629–634. doi: 10.1128/jb.52.6.629-634.1946, PMID: 16561227 PMC518248

[ref8] Chaumeil P.-A. Mussig A. J. Hugenholtz P. Parks D. H. (2020). GTDB-Tk: a toolkit to classify genomes with the genome taxonomy database. Bioinformatics 36, 1925–1927. doi: 10.1093/bioinformatics/btz848, PMID: 31730192 PMC7703759

[ref9] Choi S.-H. Kim J.-S. Park J.-E. Lee K. C. Eom M. K. Oh B. S.. (2019). *Anaerotignum faecicola* sp. nov., isolated from human faeces. J. Microbiol. 57, 1073–1078. doi: 10.1007/s12275-019-9268-3, PMID: 31680219

[ref10] Christensen L. F. García-Béjar B. Bang-Berthelsen C. H. Hansen E. B. (2022). Extracellular microbial proteases with specificity for plant proteins in food fermentation. Int. J. Food Microbiol. 381:109889. doi: 10.1016/j.ijfoodmicro.2022.109889, PMID: 36057216

[ref11] Di Tommaso P. Chatzou M. Floden E. W. Barja P. P. Palumbo E. Notredame C. (2017). Nextflow enables reproducible computational workflows. Nat. Biotechnol. 35, 316–319. doi: 10.1038/nbt.3820, PMID: 28398311

[ref12] Dieckmann M. A. Beyvers S. Nkouamedjo-Fankep R. C. Hanel P. H. G. Jelonek L. Blom J.. (2021). EDGAR3.0: comparative genomics and phylogenomics on a scalable infrastructure. Nucleic Acids Res. 49, W185–W192. doi: 10.1093/nar/gkab341, PMID: 33988716 PMC8262741

[ref13] Fonknechten N. Chaussonnerie S. Tricot S. Lajus A. Andreesen J. R. Perchat N.. (2010). *Clostridium sticklandii*, a specialist in amino acid degradation: revisiting its metabolism through its genome sequence. BMC Genomics 11:555. doi: 10.1186/1471-2164-11-555, PMID: 20937090 PMC3091704

[ref14] Greub G. (2012). Culturomics: a new approach to study the human microbiome. Clin. Microbiol. Infect. 18, 1157–1159. doi: 10.1111/1469-0691.12032, PMID: 23148445

[ref15] Grissa I. Vergnaud G. Pourcel C. (2007). CRISPRFinder: a web tool to identify clustered regularly interspaced short palindromic repeats. Nucleic Acids Res. 35, W52–W57. doi: 10.1093/nar/gkm360, PMID: 17537822 PMC1933234

[ref16] Hahnke S. Langer T. Koeck D. E. Klocke M. (2016). Description of *Proteiniphilum saccharofermentans* sp. nov., *Petrimonas mucosa* sp. nov. and *Fermentimonas caenicola* gen. Nov., sp. nov., isolated from mesophilic laboratory-scale biogas reactors, and emended description of the genus *Proteiniphilum*. Int. J. Syst. Evol. Microbiol. 66, 1466–1475. doi: 10.1099/ijsem.0.000902, PMID: 26782749

[ref17] Hassa J. Wibberg D. Maus I. Pühler A. Schlüter A. (2019). Genome analyses and genome-centered metatranscriptomics of Methanothermobacter wolfeii strain SIV6, isolated from a thermophilic production-scale biogas fermenter. Microorganisms 8:E13. doi: 10.3390/microorganisms8010013, PMID: 31861790 PMC7022856

[ref18] Jia B. Raphenya A. R. Alcock B. Waglechner N. Guo P. Tsang K. K.. (2017). CARD 2017: expansion and model-centric curation of the comprehensive antibiotic resistance database. Nucleic Acids Res. 45, D566–D573. doi: 10.1093/nar/gkw1004, PMID: 27789705 PMC5210516

[ref19] Kieliszek M. Pobiega K. Piwowarek K. Kot A. M. (2021). Characteristics of the proteolytic enzymes produced by lactic acid bacteria. Molecules 26:1858. doi: 10.3390/molecules26071858, PMID: 33806095 PMC8037685

[ref20] Koch C. Müller S. Harms H. Harnisch F. (2014). Microbiomes in bioenergy production: from analysis to management. Curr. Opin. Biotechnol. 27, 65–72. doi: 10.1016/j.copbio.2013.11.006, PMID: 24863898

[ref21] Koeck D. E. Hahnke S. Zverlov V. V. (2016a). Herbinix luporum sp. nov., a thermophilic cellulose-degrading bacterium isolated from a thermophilic biogas reactor. Int. J. Syst. Evol. Microbiol. 66, 4132–4137. doi: 10.1099/ijsem.0.001324, PMID: 27453473

[ref22] Koeck D. E. Ludwig W. Wanner G. Zverlov V. V. Liebl W. Schwarz W. H. (2015). *Herbinix hemicellulosilytica* gen. Nov., sp. nov., a thermophilic cellulose-degrading bacterium isolated from a thermophilic biogas reactor. Int. J. Syst. Evol. Microbiol. 65, 2365–2371. doi: 10.1099/ijs.0.000264, PMID: 25872956

[ref23] Koeck D. E. Mechelke M. Zverlov V. V. Liebl W. Schwarz W. H. (2016b). *Herbivorax saccincola* gen. Nov., sp. nov., a cellulolytic, anaerobic, thermophilic bacterium isolated via in sacco enrichments from a lab-scale biogas reactor. Int. J. Syst. Evol. Microbiol. 66, 4458–4463. doi: 10.1099/ijsem.0.001374, PMID: 27499077

[ref24] Köller N. Hahnke S. Zverlov V. Wibberg D. Klingl A. Busche T.. (2022). “*Anaeropeptidivorans aminofermentans* gen. Nov., sp. nov., a mesophilic proteolytic salt-tolerant bacterium isolated from a laboratory-scale biogas fermenter, and emended description of clostridium colinum”, in International Journal of Systematic and Evolutionary Microbiology. ed. M. A. Amoozegar. 10.1099/ijsem.0.005668 36748496

[ref25] Kougias P. G. Campanaro S. Treu L. Tsapekos P. Armani A. Angelidaki I. (2018). Spatial distribution and diverse metabolic functions of lignocellulose-degrading uncultured bacteria as revealed by genome-centric metagenomics. Appl. Environ. Microbiol. 84, e01244–e01218. doi: 10.1128/AEM.01244-18, PMID: 30006398 PMC6121989

[ref26] Lagier J.-C. Armougom F. Million M. Hugon P. Pagnier I. Robert C.. (2012). Microbial culturomics: paradigm shift in the human gut microbiome study. Clin Microb Infect 18, 1185–1193. doi: 10.1111/1469-0691.12023, PMID: 23033984

[ref27] Lagier J.-C. Hugon P. Khelaifia S. Fournier P.-E. La Scola B. Raoult D. (2015). The rebirth of culture in microbiology through the example of culturomics to study human gut microbiota. Clin. Microbiol. Rev. 28, 237–264. doi: 10.1128/CMR.00014-14, PMID: 25567229 PMC4284300

[ref28] Lambie S. C. Kelly W. J. Leahy S. C. Li D. Reilly K. McAllister T. A.. (2015). The complete genome sequence of the rumen methanogen Methanosarcina barkeri CM1. Stand. Genomic Sci. 10:57. doi: 10.1186/s40793-015-0038-5, PMID: 26413197 PMC4582637

[ref29] Li H. (2013). Aligning sequence reads, clone sequences and assembly contigs with BWA-MEM. doi: 10.48550/arXiv.1303.3997

[ref30] Lietzan A. D. St. Maurice M. (2014). Functionally diverse biotin-dependent enzymes with oxaloacetate decarboxylase activity. Arch. Biochem. Biophys. 544, 75–86. doi: 10.1016/j.abb.2013.10.014, PMID: 24184447

[ref31] Maus I. Klocke M. Derenkó J. Stolze Y. Beckstette M. Jost C.. (2020a). Impact of process temperature and organic loading rate on cellulolytic / hydrolytic biofilm microbiomes during biomethanation of ryegrass silage revealed by genome-centered metagenomics and metatranscriptomics. Environ. Microb. 15:7. doi: 10.1186/s40793-020-00354-x, PMID: 33902713 PMC8067321

[ref32] Maus I. Tubbesing T. Wibberg D. Heyer R. Hassa J. Tomazetto G.. (2020b). The role of Petrimonas mucosa ING2-E5AT in mesophilic biogas reactor systems as deduced from multiomics analyses. Microorganisms 8:E2024. doi: 10.3390/microorganisms8122024, PMID: 33348776 PMC7768429

[ref33] Maus I. Wibberg D. Stantscheff R. Stolze Y. Blom J. Eikmeyer F.-G.. (2015). Insights into the annotated genome sequence of Methanoculleus bourgensis MS2(T), related to dominant methanogens in biogas-producing plants. J. Biotechnol. 201, 43–53. doi: 10.1016/j.jbiotec.2014.11.020, PMID: 25455016

[ref34] Meyer F. Goesmann A. McHardy A. C. Bartels D. Bekel T. Clausen J.. (2003). GenDB--an open source genome annotation system for prokaryote genomes. Nucleic Acids Res. 31, 2187–2195. doi: 10.1093/nar/gkg312, PMID: 12682369 PMC153740

[ref500] Mumme J. Linke B. Tölle R. (2010). Novel upflow anaerobic solid-state (UASS) reactor. Bioresour. Technol. 101, 592–9. doi: 10.1016/j.biortech.2009.08.073, PMID: 19748268

[ref35] Nelkner J. Henke C. Lin T. W. Pätzold W. Hassa J. Jaenicke S.. (2019). Effect of long-term farming practices on agricultural soil microbiome members represented by metagenomically assembled genomes (MAGs) and their predicted plant-beneficial genes. Genes 10. doi: 10.3390/genes10060424, PMID: 31163637 PMC6627896

[ref36] Ondov B. D. Starrett G. J. Sappington A. Kostic A. Koren S. Buck C. B.. (2019). Mash screen: high-throughput sequence containment estimation for genome discovery. Genome Biol. 20:232. doi: 10.1186/s13059-019-1841-x, PMID: 31690338 PMC6833257

[ref37] Poehlein A. Schlien K. Chowdhury N. P. Gottschalk G. Buckel W. Daniel R. (2016). Complete genome sequence of the amino acid-fermenting clostridium propionicum X2 (DSM 1682). Genome Announc. 4, e00294–e00216. doi: 10.1128/genomeA.00294-16, PMID: 27081148 PMC4832176

[ref38] Rawlings N. D. Barrett A. J. Thomas P. D. Huang X. Bateman A. Finn R. D. (2018). The MEROPS database of proteolytic enzymes, their substrates and inhibitors in 2017 and a comparison with peptidases in the PANTHER database. Nucleic Acids Res. 46, D624–D632. doi: 10.1093/nar/gkx1134, PMID: 29145643 PMC5753285

[ref39] Seemann T. (2014). Prokka: rapid prokaryotic genome annotation. Bioinformatics 30, 2068–2069. doi: 10.1093/bioinformatics/btu153, PMID: 24642063

[ref40] Solanki P. Putatunda C. Kumar A. Bhatia R. Walia A. (2021). Microbial proteases: ubiquitous enzymes with innumerable uses. 3 Biotech 11:428. doi: 10.1007/s13205-021-02928-z, PMID: 34513551 PMC8425024

[ref41] Solden L. Lloyd K. Wrighton K. (2016). The bright side of microbial dark matter: lessons learned from the uncultivated majority. Curr. Opin. Microbiol. 31, 217–226. doi: 10.1016/j.mib.2016.04.020, PMID: 27196505

[ref42] Søndergaard D. Pedersen C. N. S. Greening C. (2016). HydDB: a web tool for hydrogenase classification and analysis. Sci. Rep. 6:34212. doi: 10.1038/srep34212, PMID: 27670643 PMC5037454

[ref43] Sorbara M. T. Littmann E. R. Fontana E. Moody T. U. Kohout C. E. Gjonbalaj M.. (2020). Functional and genomic variation between human-derived isolates of Lachnospiraceae reveals inter- and intra-species diversity. Cell Host Microbe 28, 134–146.e4. doi: 10.1016/j.chom.2020.05.005, PMID: 32492369 PMC7351604

[ref44] Stolze Y. Bremges A. Rumming M. Henke C. Maus I. Pühler A.. (2016). Identification and genome reconstruction of abundant distinct taxa in microbiomes from one thermophilic and three mesophilic production-scale biogas plants. Biotechnol. Biofuels 9:156. doi: 10.1186/s13068-016-0565-3, PMID: 27462367 PMC4960831

[ref45] Tejerizo G. T. Kim Y. S. Maus I. Wibberg D. Winkler A. Off S.. (2017). Genome sequence of *Methanobacterium congolense* strain Buetzberg, a hydrogenotrophic, methanogenic archaeon, isolated from a mesophilic industrial-scale biogas plant utilizing bio-waste. J. Biotechnol. 247, 1–5. doi: 10.1016/j.jbiotec.2017.02.015, PMID: 28216101

[ref46] Tholozan J. L. Touzel J. P. Samain E. Grivet J. P. Prensier G. Albagnac G. (1992). Clostridium neopropionicum sp. nov., a strict anaerobic bacterium fermenting ethanol to propionate through acrylate pathway. Arch. Microbiol. 157, 249–257. doi: 10.1007/BF00245158, PMID: 1510558

[ref47] Tomazetto G. Hahnke S. Wibberg D. Pühler A. Klocke M. Schlüter A. (2018). Proteiniphilum saccharofermentans str. M3/6T isolated from a laboratory biogas reactor is versatile in polysaccharide and oligopeptide utilization as deduced from genome-based metabolic reconstructions. Biotechnol. Rep. 18:e00254. doi: 10.1016/j.btre.2018.e00254, PMID: 29892569 PMC5993710

[ref48] Ueki A. Goto K. Ohtaki Y. Kaku N. Ueki K. (2017). Description of *Anaerotignum aminivorans* gen. Nov., sp. nov., a strictly anaerobic, amino-acid-decomposing bacterium isolated from a methanogenic reactor, and reclassification of clostridium propionicum, clostridium neopropionicum and clostridium lactatifermentans as species of the genus *Anaerotignum*. Int. J. Syst. Evol. Microbiol. 67, 4146–4153. doi: 10.1099/ijsem.0.002268, PMID: 28905695

[ref49] Vacca M. Celano G. Calabrese F. M. Portincasa P. Gobbetti M. De Angelis M. (2020). The controversial role of human gut *Lachnospiraceae*. Microorganisms 8:573. doi: 10.3390/microorganisms8040573, PMID: 32326636 PMC7232163

[ref50] van der Wielen P. W. J. J. Rovers G. M. L. L. Scheepens J. M. A. Biesterveld S. (2002). Clostridium lactatifermen tans sp. nov., a lactate-fermenting anaerobe isolated from the caeca of a chicken. Int. J. Syst. Evol. Microbiol. 52, 921–925. doi: 10.1099/00207713-52-3-921, PMID: 12054258

[ref51] Vital M. Howe A. C. Tiedje J. M. (2014). Revealing the bacterial butyrate synthesis pathways by analyzing (meta)genomic data. mBio 5:e00889. doi: 10.1128/mBio.00889-14 24757212 PMC3994512

[ref52] Zhang H. Yohe T. Huang L. Entwistle S. Wu P. Yang Z.. (2018). dbCAN2: a meta server for automated carbohydrate-active enzyme annotation. Nucleic Acids Res. 46, W95–W101. doi: 10.1093/nar/gky418, PMID: 29771380 PMC6031026

