# Supplementary material for: The novel oligopeptide utilizing species Anaeropeptidivorans aminofermentans M3/9T, its role in anaerobic digestion and occurrence as deduced from large-scale fragment recruitment analyses
Source: Front Microbiol. 2022 Nov 9;13:1032515. doi: 10.3389/fmicb.2022.1032515 (PMC9682168; doi:10.3389/fmicb.2022.1032515)
Supplement: Supplementary file 4 [file Data_Sheet_1.docx]

Supplementary Material

# Supplementary Figures and Tables

## Supplementary Figures

**Supplementary Figure 1:** Phylogenetic affiliation of *Anaeropeptidivorans aminofermentans* M3/9^T^. 16S rRNA-based phylogenetic tree is calculated by the Type (Strain) Genome Server (TYGS) platform. 16S rRNA sequences are derived from the TYGS taxonomy database including all validly published, genome sequenced prokaryotic species (Meier-Kolthoff et al., 2022).

**Supplementary Figure 2:** Trimmed mean of the read coverage of *Anaeropeptidivorans aminofermentans* M3/9^T^, *Anaerotignum faecicola* DSM 107953^T^ and *Anaerotignum lactatifermentans* DSM 14214^T^ genomes which are ≥95% covered by metagenome sequences.

## Supplementary Tables

**Supplementary Table 1:** List with 477,981 metagenomic data sets of the Sequence Read Archive (SRA) Mirror of the de.NBI Cloud (https://www.denbi.de/cloud).

**Supplementary Table 2:** *A. aminofermentans* M3/9^T^ genes predicted to be involved in central fermentation metabolism.

**Supplementary Table 3:** *A. aminofermentans* M3/9^T^ genes predicted to encode peptidases as deduced from the comparison against the MEROPS database (Rawlings et al., 2018).
