# Supplementary figures and images for: The novel oligopeptide utilizing species Anaeropeptidivorans aminofermentans M3/9T, its role in anaerobic digestion and occurrence as deduced from large-scale fragment recruitment analyses
Source: Front Microbiol. 2022 Nov 9;13:1032515. doi: 10.3389/fmicb.2022.1032515 (PMC9682168; doi:10.3389/fmicb.2022.1032515)

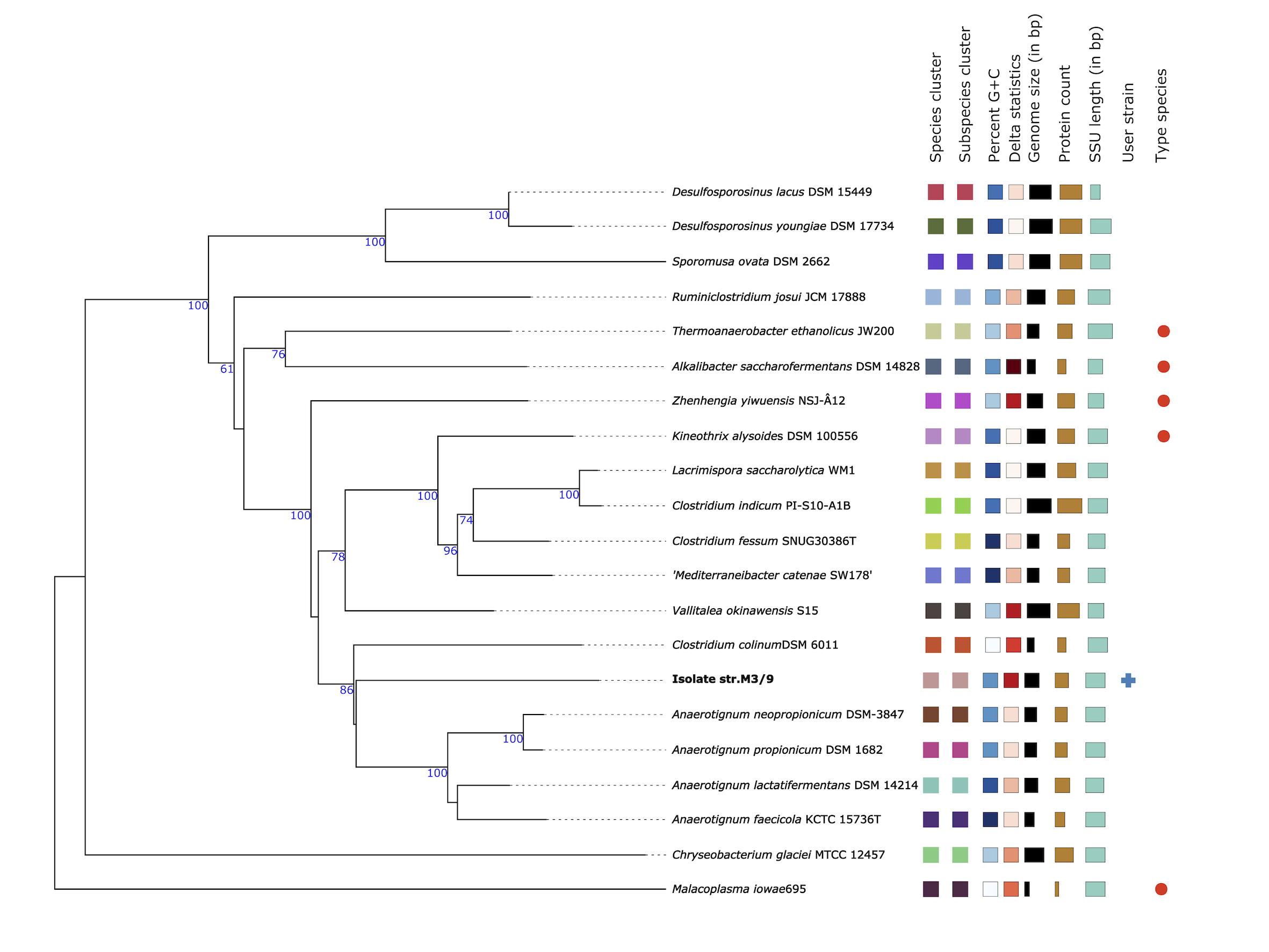

Supplement: Supplementary file 5 [file Image_1.TIFF]

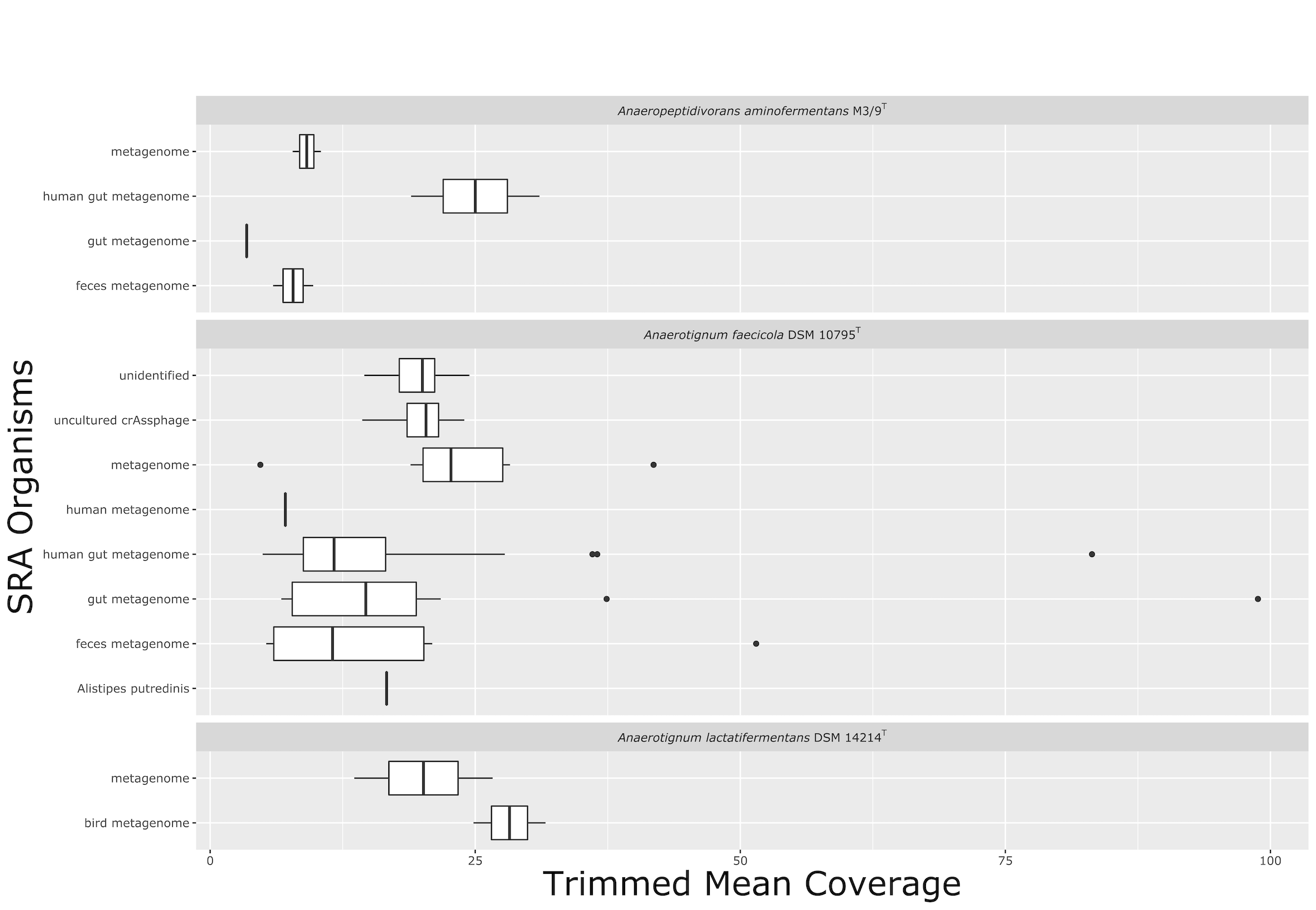

Supplement: Supplementary file 6 [file Image_2.TIFF]
